# Inventory of molecular markers affecting biological characteristics of avian influenza A viruses

**DOI:** 10.1007/s11262-019-01700-z

**Published:** 2019-08-19

**Authors:** Annika Suttie, Yi-Mo Deng, Andrew R. Greenhill, Philippe Dussart, Paul F. Horwood, Erik A. Karlsson

**Affiliations:** 1 Virology Unit, Institut Pasteur du Cambodge, Institut Pasteur International Network, 5 Monivong Blvd, PO Box #983, Phnom Penh, Cambodia; 2 School of Health and Life Sciences, Federation University, Churchill, Australia; 3 World Health Organization Collaborating Centre for Reference and Research on Influenza, Peter Doherty Institute for Infection and Immunity, Melbourne, Australia; 4 College of Public Health, Medical and Veterinary Sciences, James Cook University, Townsville, QLD Australia

**Keywords:** Avian influenza, Influenza virus, Molecular marker, Mutation, Risk assessment, Molecular inventory

## Abstract

Avian influenza viruses (AIVs) circulate globally, spilling over into domestic poultry and causing zoonotic infections in humans. Fortunately, AIVs are not yet capable of causing sustained human-to-human infection; however, AIVs are still a high risk as future pandemic strains, especially if they acquire further mutations that facilitate human infection and/or increase pathogenesis. Molecular characterization of sequencing data for known genetic markers associated with AIV adaptation, transmission, and antiviral resistance allows for fast, efficient assessment of AIV risk. Here we summarize and update the current knowledge on experimentally verified molecular markers involved in AIV pathogenicity, receptor binding, replicative capacity, and transmission in both poultry and mammals with a broad focus to include data available on other AIV subtypes outside of A/H5N1 and A/H7N9.

## Introduction

Avian influenza viruses (AIVs) (Family: *Orthomyxoviridae*, genus: *Influenzavirus A*) are negative sense, single-stranded RNA viruses found globally in their natural reservoir hosts, wild waterfowl, and other aquatic birds (mainly of the orders *Anseriformes* and *Charadriiformes*). AIV genomes are composed of eight genomic RNA segments encoding at least twelve viral proteins. Viral nomenclature is based on combinations of the two surface glycoproteins, hemagglutinin (HA) and neuraminidase (NA). To date, sixteen HA (H1–H16) and nine NA (N1–N9) subtypes circulate in wild aquatic birds [1], and two novel HA subtypes (H17 and H18) and two novel NA subtypes (N10 and N11) have recently been identified in bats [2–6]. AIVs are sporadically transmitted from waterfowl to domestic avian species, resulting in a number of stable AIV lineages in domestic poultry. These domestic lineages typically circulate in poultry flocks as low pathogenic (LPAIV) variants, causing little to no apparent illness; however, some subtypes (namely A/H5 and A/H7) have the potential to mutate to form highly pathogenic (HPAIV) variants capable of causing high mortality rates in domestic avian species. These HPAIV lineages then spread back to wild bird species, potentiating global spread [1].

Occasionally, AIVs spillover into mammalian species such as humans, swine, marine mammals, and various members of the Equidae, Felidae, and Canidae families [7–10], generally through close contact with domestic poultry, the primary intermediate host [11]. These spillovers sometimes result in stable, mammalian-adapted lineages as have occurred with subtype A/H3N8 AIVs in equines and canines [9]. A number of zoonotic infections with different AIV subtypes have occurred in humans, including: A/H5 (H5N1,H5N6), A/H6N1, A/H7 (H7N2, H7N3, H7N4, H7N7, H7N9), A/H9N2, and A/H10 (H10N7, H10N8) [12]. Clinical symptoms vary in severity from asymptomatic infections, to conjunctivitis or influenza-like-illness, to severe acute respiratory illness. Infections with particularly virulent viral subtypes, such as A/H5 and A/H7, or infections in immunocompromised, high-risk hosts can cause high morbidity, respiratory distress and/or multiple-organ failure, and, in some cases, mortality [13]. Fortunately, minimal human-to-human transmission occurs in human AIV cases; however, each zoonotic event in mammals represents a risk for AIVs to adapt replicative and transmissible properties in the mammalian host. Indeed, several studies using ferret models have shown that these AIVs have the potential to mutate into airborne transmissible forms, potentially with some mutations already present in nature [14, 15]. However, while these observations are very important, point mutations associated with airborne transmission between ferrets are not universal, as they may not confer similar changes in biological characteristics of these viruses in other mammalian species, including humans.

Given the continual circulation of AIVs in wild birds and domestic poultry, the potential for human spillover, and the mutable nature of AIV itself, it is paramount to understand the potential risk of any emerging AIV. Indeed, risk assessment tools are vital in pandemic preparedness planning [16]. Genetic variation resulting in changes in viral properties such as receptor binding, replicative capacity, and transmission is a critical component of risk assessment. Historically, assessing these factors occurs in vivo; however, the ability to evaluate these properties in silico from sequence data allows for faster, more efficacious assessment of novel, emerging strains. Numerous studies identify molecular markers for AIV risk, and several previous papers compile markers of interest. Here, we summarize and update the current knowledge on experimentally verified molecular markers affecting biological characteristics of avian influenza viruses important for risk assessment and broaden the scope outside of the A/H5N1 and A/H7N9 subtypes.

## Methodology

### Data collection

All available information on AIV molecular markers/mutations was collected from: the CDC H5N1 Genetic changes inventory [17], the WHO Working Group on Surveillance of Influenza Antiviral Susceptibility (WHO-AVWG) [18], and publications summarizing AIV mutations and molecular markers affecting biological characteristics and potential risk [19–21]. Journal articles were sourced for each specific subtype using PubMed searches with MeSH Terms, Boolean operators and wild cards. For example, terms used to search for mutations in H6 AIVs: (influenza A virus[MeSH Terms]) AND (mutation OR mutagenesis OR virulence[MeSH Terms]) AND (H6[Title/Abstract] OR H6N*[Title/Abstract]).

### Inclusion/exclusion

All mutations/molecular markers from influenza viruses of avian origin were included in the initial screening and tabulation, including viruses isolated from humans AIV cases. However, we do not consider this inventory as an exhaustive list. Several studies computationally predict molecular markers of viral adaptation to humans [22–26]. While this work is extremely valuable, experimental validation of the majority of the markers described in these studies is not available and, therefore, necessitated exclusion from this data summary. In addition, since this inventory focuses on AIV and zoonotic infection, genome mutations in human seasonal influenza viruses were also excluded. As several publications observe the same mutations causing similar biological characteristics that could indicate risk markers, we excluded duplicate information from the tables.

### Numbering

For all data presented, HA mutations are numbered according to the H3 subtype to maintain consistency with available literature; however, H5 numbering was also included in the table. N2 numbering was used for NA as this is most commonly used in the current literature. Internal proteins (including deletions) and deletions in NA are numbered according to the full length of A/Goose/Guangdong/1/1996 genome segments.

## Surface proteins

### Hemagglutinin (HA; Table 1)

Hemagglutinin is a homotrimeric transmembrane protein and is the most abundant protein present on the surface of influenza virions. For virions to successfully enter and replicate in host cells, host proteases cleave the HA0 precursor into two subunits, HA1 and HA2. The main role of the HA1 subunit is to initiate infection by recognizing and binding receptors on the host cell surface. After internalization and entrance into the endosomal pathway, the HA2 subunit fuses the viral and endosomal membranes, creating a pore for viral RNA entry into the host cell, and initiation of transcription and translation of viral products [27, 28]. Several mutations in HA are associated with changes in viral fitness and transmissibility, as they affect viral receptor binding avidity/specificity or viral membrane fusion activities (Table 1).

**Table 1:** Molecular markers/motifs in the hemagglutinin (HA; segment 4) gene of influenza virus experimentally verified molecular markers involved in AIV pathogenicity, receptor binding, replicative capacity, and transmission in both poultry and mammals

Mutation/motif		Phenotype	Subtypes tested	References
H3 numbering^a^	H5 numbering^b^			
D101N	D94N	Increased virus binding to α2–6	H5N1	[200]
S126N	S121N	Increased virus binding to α2–6	H5N1	[201]
S137A	S133A	Increased pseudovirus binding to α2–6	H5N1	[44]
A138V	A134V	Increased infectivity in SIAT Cells	H5N1	[202, 203]
G143R	G139R	Increased virus binding to α2–6	H5N1	[204]
S158N	S154N	Increased virus binding to α2–6	H5N1	[201]
N158D	N154D	Decreased virulence in mice	H9N2	[205]
S159N	S155N	Increased virus binding to α2–6	H5N1	[201]
T160A	T156A	Increased virus binding to α2–6, increased transmission in guinea pigs	H5N1	[201, 206]
G186V	G182V	Increased virus binding to α2–3	H7N9	[207]
N186K/D	N182K/D	Increased virus binding to α2–6	H5N1	[201, 203, 204, 206]
V186N	V182N	Increased binding to α2–6, decreased binding to α2–3	H13N6	[208]
P186L	P182L	Decreased binding to α2–3	H6N1	[209]
D187G	D183G	Increased virus binding to α2–6	H5N1	[47]
E190G	E186G	Increased virus binding to α2–6, maintained α2–3 binding, decreased virulence in mice	H5N1	[47, 210]
E190V	E186V	Decreased binding to α2–3 and α2–6	H6N2	[211]
T190V	T186V	Enhances binding affinity to mammalian cells and replication in mammalian cells	H9N2	[212]
T192I	T188I	Increased pseudovirus binding to α2–6	H5N1	[44]
K193R/T	K189R/T	Increased virus binding to α2–6	H5N1	[201, 213]
Q196R/H	Q192R/H	Increased virus binding to α2–6	H5N1	[47, 204, 214]
N197K	N193K	Increased virus binding to α2–6	H5N1	[204]
V214I	V210I	Increased virus binding to α2–6	H5N1	[214]
G225D	G221D	Increased virus binding to α2,6	H6N1	[43]
Q226L	Q222L	Increased virus binding to α2–6	H4N6	[50]
		Increased virus binding to α2–6	H6N2	[211]
		Increased virus binding to α2–6, decreased binding to α2–3	H5N1	[46]
		Increased virus binding to α2–3, decreased binding to α2–6	H7N9	[52, 215]
		Increased virus binding to α2–6, enhanced replication in mammalian cells and ferrets, enhanced contact transmission in ferrets	H9N2	[49, 54]
		Loss of binding to α2–3	H10N8 (human isolate)	[53, 216, 217]
L226I	L222I	Decreased binding to α2–3	H7N9	[52]
S227N	S223N	Increased virus binding to α2–6	H5N1	[46, 47, 203, 218, 219]
G228A/S	G224A/S	Increased binding to α2–6, dual receptor specificity	H4N6	[50]
G228S	G224S	Increased viral replication in mammalian cells and virulence in mice	H1N2	[220]
		Increased virus binding to α2–6	H5N1	[45, 46, 201, 209]
		Decreased virus binding to α2–3	H6N2	[211]
		Decreased binding to α2–3 and α2–6 receptors	H7N9	[51]
		Decreased binding to α2–3, no binding to α2–6	H10N8 (human isolate)	[53, 217]
P239S	P235S	Increased virus binding to α2–6	H5N1	[214]
E255K	E251K	Increased virus binding to α2–6	H5N1	[47]
326 to 329	323 to 330 (R-X-R, K-R)	Polybasic cleavage motif sequence required for high pathogenicity avian influenza viruses	H5Nx	[221–228]
			H7Nx	[229–231]
K387I	K388I	Decreased pH of fusion, increased HA stability, increased replication efficiency and virulence in mice	H5N1	[130, 232, 233]
K393E	K394E	Increased pH of fusion, decreased HA stability, decreased virulence in mice	H7N9	[234]
E83K, S128P	E75K, S123P	Increased virus binding to α2–6	H5N1	[204]
E83K, S128P, R496K	E75K, S123P, R497K	Increased virus binding to α2–6	H5N1	[204]
E83K, S128P, N197K, R496K	E75K, S123P, N193K, R497K	Increased virus binding to α2–6	H5N1	[204]
E83K, N197K	E75K, N193K	Increased virus binding to α2–6	H5N1	[204]
E83K, N197K, R496K	E75K, N193K, R497K	Increased virus binding to α2–6	H5N1	[204]
E83K, R496K	E75K, R497K	Increased virus binding to α2–6	H5N1	[204]
H110Y, T160A, Q226L, G228S	H103Y, T156A, Q222L, G224S (with PB2: E627K; PB1: H99Y)	Airborne transmissible in ferrets	H5N1	[15, 58]
S114R, T115I	S107R, T108I	Increased virulence in chickens and mice, increased pH of fusion	H5N1	[235]
S128P, N197K	S123P, N193K	Increased virus binding to α2–6	H5N1	[204]
S128P, N197K, R496K	S123P, N193K, R497K	Increased virus binding to α2–6	H5N1	[204]
S128P, R496K	S123P, R497K	Increased virus binding to α2–6	H5N1	[204]
∆^c^, A138V	L129V, A134V	Increased virus binding to α2–6	H5N1	[236]
∆, I155T	L129del, I151T	Increased virus binding to α2–6	H5N1	[214, 236]
S137A, T192I	S133A, T188I	Increased pseudovirus binding to α2–6	H5N1	[44]
G143R, N186K	G139R, N182K	Decreased binding to α2–3, increased virus binding to α2–6	H5N1	[46, 204]
N158D, N224K, Q226L, T318I	N154D, N220K, Q222L, T315I	Transmissible among ferrets	H5N1	[14]
N158S, Q226L	N154S, Q222L	Increased virus binding to α2–6	H5N1	[237]
N158S, Q226L, N248D	N154S, Q222L, N244D	Increased virus binding to α2–6	H5N1	[237]
S159N, T160A	S155N, T156A	Increased virus binding to α2–6	H5N1	[201, 209]
S159N, T160A, S227N	S155N, T156A, S223N	Increased virus binding to α2–6, reduced lethality and systemic spread in mice	H5N1	[238]
T160A, K193T, N224K, Q226L	T156A, K189T, N220K, Q222L	Increased virus binding to α2–6	H5N1	[213]
T160A, Q226L	T156A, Q222L	Increased virus binding to α2–6	H5N1	[201, 209]
T160A, Q226L, G228S	T156A, Q222L, G224S	Increased virus binding to α2–6	H5N1	[201, 209, 239]
T160A, S227N	T156A, S223N	Increased virus binding to α2–6	H5N1	[201, 209]
V186N, N228K	V182N, N224K	Increased virus binding to α2–6	H7N9	[51]
V186K/G, K193T, G228S	V182 K/G, K189T, G224S	Increased virus binding to α2–6	H7N9	[51]
V186N, N224K, G228S	V182N, N220K, G224S	Increased virus binding to α2–6	H7N9	[51]
N186K, Q196R, Q226L, S227N, G228S	N182K, Q192R, Q222L, S223N, G224S	Increased virus binding to α2–6	H5N1	[46]
N186K, Q226L, S227N, G228S	N182K, Q222L, S223N, G224S	Increased virus binding to α2–6	H5N1	[46]
N186K, Q226L, G228S	N182K, Q222L, G224S	Increased virus binding to α2–6	H5N1	[46]
E187G, E190D, K193S, Q226L, G228S	E183G, E186D, K189S, Q222L, G224S	Increased virus binding to α2–6	H5N1	[240]
E187G, Q226L,G228S	E183G, Q222L,G224S	Increased virus binding to α2–6	H5N1	[47]
D187G, S227N	D183G, S223N	Increased virus binding to α2–6	H5N1	[47]
T189A, G192R	T185A, G188R	Enhanced replication in ferrets, transmitted via aerosols among ferrets	H9N2 (with human H3N2 backbone)	[241]
E190G, Q226E, G228S	E186G, Q222E, G224S	Increased virus binding to α2–6	H5N1	[47]
K193T, G228S	K189T, G224S	Dual α2–3 and α2–6 binding	H7N9	[51]
K193R, Q226L, G228S	K189R, Q222L, G224S	Increased virus binding to α2–6	H5N1	[239, 240]
Q196R, Q226L, S227N, G228S	Q192R, Q222L, S223N, G224S	Increased virus binding to α2–6	H5N1	[46]
Q196R, Q226L, G228S	Q192R, Q222L, G224S	Increased virus binding to α2–6	H5N1	[46, 47]
Q196R, S227N	Q192R, S223N	Increased virus binding to α2–6	H5N1	[46, 47]
N197K, R496K	N193K, R497K	Increased virus binding to α2–6	H5N1	[204]
K222Q, S227R	K218Q, S223R	Increased virus binding to α2–3 and α2–6	H5N1	[242]
N224K, G228S	N220K, G224S	Increased virus binding to α2–6	H7N9	[51]
Q226L, S227N, G228S	Q222L, S223N, G224S	Increased virus binding to α2–6	H5N1	[46]
Q226L, G228S	Q222L, G224S	Increased virus binding to α2–6	H4N6	[50]
		Increased virus binding to α2–6; decreased antiviral response in host; reduced tissue tropism in guinea pigs	H5N1	[45–47, 201, 206, 209, 215, 237, 239, 240, 243, 244]
		Increased virus binding to α2–6	H7N7 (human isolate)	[245]
		Loss of binding to α2–3, no gain of binding to α2–6	H10N8 (human isolate)	[53, 216, 217]

Single mutations are presented first followed by motifs involving multiple mutations. Individual markers and motifs are listed in numerical order for ease of identifying mutation of interest

^a^Mutation/location H3 numbering relative to A/Aichi/2/1968 (H3N2)

^b^Mutation/location H5 numbering relative to A/Vietnam/1203/2004 (H5N1)

^c^∆ indicates amino acids present in A/H5 but are deleted compared to A/H3

#### Receptor binding specificity

The HA protein initiates influenza virus infections by recognizing and binding to sialylated glycans on the surface of host cells. Distribution and type of sialic acid residues in the host respiratory tract and the receptor binding preference of AIVs are major determinants of viral host range and transmissibility [29–32]. AIVs typically bind to sialic acids in α2,3 linkage to galactose, whereas human adapted viruses bind sialic acids in α2,6 linkage. While the respiratory tract of humans contains both linkages of sialic acids, α2,6 linkages are more abundant on the surface of epithelial cells lining the upper respiratory tract, allowing for the spread of human adapted viruses through the production of aerosols by sneezing and coughing [33, 34]. α2,3 linkages are mainly found in the lower respiratory tract of humans, permitting infection with AIVs but restricting transmission [35]. In contrast, avian species have both α2,6 and α2,3 linked sialic acids in their respiratory and intestinal tracts, though abundance and distribution differs between species [36–39]. Generally, epithelial cells with α2,3 linkages are more abundant in the avian intestinal tract, facilitating transmission via the fecal–oral route.

A switch in binding preference from α2,3 “avian-type” receptors to α2,6 “human-type” receptors is considered a key factor for pandemic potential of AIVs. There are numerous HA mutations that, individually or in combination, affect viral receptor binding preference, pathogenicity and transmissibility [40]. Two mutations, E190D and G225D, increase the preference for “human-type” receptors in both the 1918, “Spanish” A/H1N1 pandemic virus and the 2009 A/H1N1 “Swine Flu” pandemic virus [41, 42]. However, the impact of these mutations on other AIVs appears to be subtype specific [43–45]. A single G225D mutation alters the receptor preference of an A/H6N1 virus isolated from a human in Taiwan to bind α2,6 human-type receptors [43] In contrast, while the same single substitution in A/H5 viruses does not increase affinity for α2,6 glycans [44–46], E190G produces a dual α2,3/α2,6 receptor binding phenotype [47]. Double E190D/G225D mutants have minimal binding to either α2,3 or α2,6 glycans in both A/H5 and A/H6 subtypes [43–45].

Unlike the 1918 and “Swine Flu” pandemic viruses, the 1957 “Asian” pandemic A/H2N2 virus and the 1968 “Hong Kong” A/H3N2 pandemic virus gained dual Q226L/G228S mutations [31, 48]. Both Q226L and G228S mutations have a profound effect on the HA receptor specificity for a large range of AIV subtypes. Individually, Q226L decreases, or completely negates, HA affinity for α2,3 “avian-type” receptors in several subtypes, namely A/H4, A/H5, A/H7, A/H9, and A/H10 [46, 49–53]. However, the effect of Q226L on α2,6 binding preference varies. In A/H5, A/H7, and A/H10 subtype AIVs, Q226L either mildly enhances, or does not influence, α2,6 binding [45, 46, 52, 53]. Conversely, Q226L substantially increases HA preference for α2,6 “human-type” receptors in A/H4 and A/H9 subtype AIVs [49, 50, 54]. In contrast to Q226L, a singular G228S mutation in A/H5 subtype isolates produces a dual binding phenotype, increasing α2,6 binding and maintaining α2,3. Therefore, the combination of Q226L/G228S dual mutations decreases, or even ablates, α2,3 binding while simultaneously increasing affinity for α2,6. However, this elegant switch does not hold true for all subtypes. For instance, single Q226L or dual Q226L/G228S mutations in a human A/H10N8 isolate significantly decrease α2,3 binding with only a minimal increase in α2,6 affinity [33, 34]. In addition, a number of other HA mutations, individually or in combination, affect viral receptor binding preference, pathogenicity and transmissibility, and more work is necessary to understand the risk of these mutations in all subtypes of concern.

Overall, the variable effect of HA mutations on AIV receptor binding preference between different AIV subtypes likely occurs due to differences in the HA receptor binding site (RBS). Four key structural elements are present in the HA RBS of all AIV subtypes: the 130-loop, 150-loop, 190-helix, and 220-loop. Conformation and amino acid composition of these structures is a primary determinant of HA receptor specify. Therefore, the variability of RBS loop length and amino acid composition between AIV subtypes could account for the variable effects observed with the same mutations in the RBS [40].

#### pH of fusion and HA stability

HA stability, or pH of fusion, refers to the pH required to trigger an irreversible conformational change in the HA1/HA2 trimer that activates the HA2 fusion peptide to mediate the fusion of the viral and endosomal membranes. This fusion creates pores through which viral ribonucleoproteins (RNPs) can exit the endosome, initiating AIV infection in the cytoplasm of the host cell. Following internalization, the endosome progressively becomes more acidic until the contents are destroyed in the host lysosome [55]. Therefore, pH of fusion dictates the efficient timing of the release of viral RNPs. If released too early, host cell recognition of viral products heightens host antiviral response, attenuating viral infection [56, 57]. If released too late, endosomal contents are destroyed, preventing release of viral products. The optimal pH of fusion varies substantially. In avian species, optimal pH of fusion can differ dramatically; however, a pH of fusion above 5.5 is believed to enhance AIV replication and transmission. In humans and ferrets, increased stability with a pH of fusion of less than 5.5 favors replication [28].

Understanding mutations affecting HA stability is vitally important since pH of fusion may partly contribute to the ability of AIVs to transmit by aerosol droplet in the ferret model [14, 15]. In subtype A/H5, a H103Y mutation stabilizes the HA, possibly increasing transmissibility [58]. A separate mutation, K387I, decreases the pH of fusion, increasing viral replication efficiency and virulence in mice while attenuating virulence in ducks [59]. A comprehensive list of mutations affecting HA stability can be found in publications by Russell [60] and Mair et al. [61]; however, the overall biological significance of pH of fusion requires further investigation. Therefore, mutations that affect pH stability without experimentally verified effects on viral replication efficiency, transmissibility, or pathogenicity have not been included in the inventory.

### Neuraminidase (NA; Table 2)

#### Viral infectivity and release from host cells

Neuraminidase (NA) is the second major transmembrane protein present on the influenza virus surface [62]. NA is a sialidase, cleaving sialic acid from glycoproteins, which enables virions to move through the mucus lining epithelial cells to initiate viral infections [63, 64], as well as mediating the release of progeny virions from the surface of infected cells [65]. HA and NA need to operate in equilibrium for efficient viral replication (reviewed in [66]) and perturbations can result in decreased viral infectivity and replication. For instance, a reduction in NA expression on seasonal human influenza viruses by experimentally introduced mutations in the viral promoter decreases virulence in mice [67]. Additionally, deletions in the NA stalk domain, the area between the enzymatically active head and the hydrophobic, envelope region, inhibit sialidase activity of NA, and alter HA binding and viral infectivity [68–70]. NA stalk length varies considerably between AIVs and a shortening of this domain is associated with adaptation of AIVs from wild birds to domestic poultry [71, 72]. A nineteen amino acid NA stalk deletion is commonly observed in highly pathogenic A/H5N1 viruses, and this deletion associates with enhanced replication capacity in mice [53, 54]. Similarly, experimentally introduced NA stalk deletions in A/H1N1, A/H7N1, A/H9N2 and A/H7N9 subtypes increase viral pathogenicity in mice and/or chickens [71, 73–78].

**Table 2:** Experimentally verified molecular markers/motifs in the neuraminidase (NA; segment 6) gene of avian influenza viruses associated with enhanced virulence and antiviral resistance

Mutation/motif (N2 numbering^a^)	Phenotype	Subtypes tested	References
49–68 deletion^b^	Enhanced virulence in mice	H5N1 (with H1N1 backbone)	[73]
			[74]
	Enhanced virulence in mice	H7N9 (human isolate)	[75]
54–72 deletion	Enhanced virulence in mice but not chickens	H5N1	[72]
	Enhanced virulence in mice	H7N9 (human isolate)	[75]
	Enhanced virulence in mice and chickens	H1N1 (avian)	[71]
54–73 deletion	Enhanced virulence in mice	H7N9 (human isolate)	[75]
54–75 deletion	Enhanced virulence in chickens but not ducks	H7N1	[76]
54–81 deletion	Enhanced replication in chicken, but not duck, cell line. Enhanced replication in respiratory tract of chickens.	H2N2	[246]
57–65 deletion	Enhanced virulence in mice	H7N9 (human isolate)	[77]
V116A	Reduced susceptibility to oseltamivir and zanamivir	H5N1	[247, 248]
I117T	Reduced susceptibility to oseltamivir and zanamivir	H5N1	[249]
E119A	Reduced susceptibility to zanamivir	H4N2	[250]
	Reduced susceptibility to oseltamivir and zanamivir	H5N1	[251, 252]
	Reduced susceptibility to oseltamivir, zanamivir, peramivir, and laninamivir	H7N9	[253]
E119D	Reduced susceptibility to zanamivir	H4N2	[250]
	Reduced susceptibility to oseltamivir, zanamivir, and peramivir	H5N1	[251]
	Reduced susceptibility to oseltamivir, zanamivir, peramivir, and laninamivir	H7N9	[253]
	Reduced susceptibility to zanamivir	H9N2	[254]
E119G	Reduced susceptibility to zanamivir	H4N2	[250]
	Reduced susceptibility to zanamivir and peramivir	H5N1	[251]
	Reduced susceptibility to zanamivir, peramivir, and laninamivir	H7N9	[253]
Q136L	Reduced susceptibility to oseltamivir and zanamivir	H5N1	[255]
	Reduced susceptibility to zanamivir, permaivir, and laninamivir	H7N9	[253]
R152K	Reduced susceptibility to laninamivir	H7N9	[253]
D198G	Reduced susceptibility to oseltamivir and zanamivir	H5N1	[256]
I222M	Reduced susceptibility to oseltamivir	H5N1	[256]
I222K	Reduced susceptibility to oseltamivir, zanamivir, peramivir, and laninamivir	H7N9	[253]
I222R	Reduced susceptibility to oseltamivir and laninamivir	H7N9	[253]
S246N	Reduced susceptibility to oseltamivir	H5N1	[248]
T247P	Reduced susceptibility to oseltamivir and zanamivir	H7N9	[253]
H274Y	Reduced susceptibility to oseltamivir and peramivir	H5N1	[251–253, 256–258]
	Reduced susceptibility to oseltamivir	H7N9	[253]
E276D	Reduced susceptibility to oseltamivir, zanamivir, peramivir, and laninamivir	H7N9	[253]
E277Q	Reduced susceptibility to oseltamivir	H5N1	[259]
R292K	Reduced susceptibility to zanamivir	H4N2	[250]
	Reduced susceptibility to oseltamivir	H6N2	[260]
	Reduced susceptibility to oseltamivir, zanamivir, peramivir, and laninamivir	H7N9	[261, 262]
	Reduced susceptibility to oseltamivir	H9N2	[254]
N294S	Reduced susceptibility to oseltamivir, zanamivir, and peramivir	H5N1	[251, 252, 257, 263]
	Reduced susceptibility to zanamivir	H7N9	[253]
R371K	Reduced susceptibility to oseltamivir, zanamivir, peramivir, and laninamivir	H7N9	[253]
A401T	Increased virus binding to α2–3	H7N9	[264]
K432T	Reduced susceptibility to zanamivir	H5N1	[265]
I117V, I314V	Reduced susceptibility to oseltamivir	H5N1	[247]
E119V, E222V	Reduced susceptibility to oseltamivir	H7N9	[266]
E119A/D/G, H274Y	Reduced susceptibility to oseltamivir, zanamivir, and peramivir	H5N1	[251]
I222L, S246N	Reduced susceptibility to oseltamivir	H5N1	[265]
I222M/V, H274Y	Reduced susceptibility to oseltamivir and peramivir	H5N1	[256]
K150N, I222L, S246N	Reduced susceptibility to oseltamivir	H5N1	[248]

Single mutations are presented first followed by motifs involving multiple mutations. Individual markers and motifs are listed in numerical order for ease of identifying mutation of interest

^a^Mutation/location N2 numbering relative to A/Aichi/2/1968 (H3N2)

^b^Deletions are numbered according to alignments with A/Goose/Guangdong/1/1996 (H5N1)

#### Antiviral susceptibility and resistance

The function of NA is essential for productive AIV infection, as exemplified by conserved NA catalytic sites across influenza virus strains. Developed as antivirals in the 1990s, neuraminidase inhibitors (NAIs) bind to this active site of NA and prevent the release of new viruses from the surface of the infected host cell. While these antiviral compounds have been used successfully for several decades, the prevalence of NAI resistance is increasing [79]. Environmental contamination with NAIs is also a recent concern, increasing the possibility of NAI resistance in AIVs from wild birds and domestic poultry [80]. The majority of NA mutations in this updated inventory are markers associated with resistance to the major NAIs currently in use globally: oseltamivir, zanamivir, laninamivir, and peramivir. The WHO-AVWG periodically releases a comprehensive table of mutations shown to affect NAI susceptibility in seasonal human influenza viruses (including both A and B genera viruses) as well as in subtype A/H5N1 and A/H7N9 AIVs [18]. The NA table (Table 2) provides a summary of WHO-AVWG NAI susceptibility markers for A/H5N1 and A/H7N9 viruses.

## Proteins of the ribonucleoprotein complex: PB2, PB1, PA, and NP

Inside the viral envelope, the eight genomic viral RNA (vRNA) segments of influenza A viruses form part of the viral ribonucleoprotein complex (vRNP). The vRNP consists of vRNA associated with multiple copies of the nucleoprotein (NP), and an RNA-dependent RNA polymerase sub-complex (RdRP), formed by polymerase basic protein 2 (PB2), polymerase basic protein 1 (PB1), and the polymerase acidic protein (PA). Cryo-EM and crystal structures of the RdRP show that PB1 forms the core of the structure, associating with PA via its N-terminal and PB2 via it’s C-terminal [81–83]. Subunits of the RdRP associate with the 5′ and 3′ ends of viral RNA and with NP. The RdRP is crucial for viral transcription and replication, producing vRNA, complementary RNA and viral messenger RNA (mRNA) (reviewed in [84, 85]). The synthesis of viral mRNA is dependent on RdRP cap snatching. Whereby, PB2 binds the 5′ cap of host RNA polymerase II transcripts [86–89], 10–13 nucleotides are cleaved by the endonuclease site of PA, and PB1 uses the cleaved fragment as a primer to initiate transcription [90]. Cap snatching not only facilitates the translation of viral mRNA, it also inhibits the production of host mRNA, referred to as host shutoff [91, 92]. Mutations that hinder AIV cap snatching ability affinity of vRNP proteins can affect viral replicative capacity and, consequently, AIV virulence.

Mutations that increase the polymerase activity of AIVs are important for the adaptation of AIVs to mammalian hosts [84]. In a number of studies, the polymerase activity of AIVs is impaired in mammalian cell lines [93]. This reduction in polymerase activity limits the transcription of viral RNA resulting in less viral material available to be packaged into progeny viruses. Additionally, limited replication capacity reduces viral genomic mutation, hindering the ability of AIVs to create progeny with beneficial mutations. A number of mutations in proteins of the polymerase complex enhance the replicative capacity of AIVs in mammalian cells. However, these increases in polymerase activity and/or replicative capacity do not always correlate with an increase in viral pathogenicity [94].

### Polymerase basic protein 2 (PB2; Table 3)

Mutations in the PB2 protein, namely one mutation—the substitution of glutamate with lysine at position 627 (E627K)—are, by far, the best-known mutations in the polymerase complex proteins to be associated with increases in viral fitness [95]. Avian viruses typically have glutamate at this position and the substitution with lysine arises during adaptation to replication in mammalian species and supports transmission [96, 97]. E627K increases viral polymerase activity and replication in mammalian cell lines at temperatures similar to those of the upper respiratory tract of humans (~ 33 °C), a trait that increases transmissibility of AIVs in mammals [98–100]. This mutation increases virulence of A/H5N1, A/H6N1, A/H7N7, A/H7N9, and A/H9N2 AIVs in mammalian models of disease [101–105]. In contrast, E627K decreases, or has no effect, on AIV virulence in chickens [106–108]. Interestingly, position 627 is situated on the surface of PB2 [84, 95, 109], and, therefore, mutations at this position influence the association of PB2 with interacting partners e.g., NP, importin α, and ANP32A [110–112]. However, the exact mechanism behind the influence of position 627 on AIV host range remains unknown. Indeed, other mutations in PB2 aside from E627K include, but are not limited to: T271A, K526R, A588V, Q591K, E627V, D701N, D701V, and S714R. All of these mutations increase viral polymerase activity in mammalian cell lines for multiple AIV subtypes. Combinations of these mutations, such as E627K/D701N/S714R, can also further enhance viral polymerase activity, replication and virulence [102].

**Table 3:** Experimentally verified molecular markers/motifs in the polymerase basic protein 2 (PB2; segment 1) gene of avian influenza virus associated with polymerase activity, virulence, and transmissibility

Mutation/motif	Phenotype	Subtypes tested	References
D9N^a^	Increased virulence in mice	H5N1	[115, 116]
V25A	Increased virulence in mice	H5N1 backbone with H1N1 NS	[267]
I63T	Decreased pathogenicity in mice	H5N1	[72]
E158G	Increased polymerase activity in mammalian cell line, increased virulence in mice	H5N2	[268]
		H5N9	[268]
E158K	Increased polymerase activity and replication in mammalian cell line, increase virulence in mice	H4N6	[269]
E192K	Increased polymerase activity in mammalian and avian cell line, increased virulence in mice	H5N1	[117]
A199S	Increased virulence in mice	H5N1	[116]
D253N	Increased polymerase activity in mammalian cell line	H9N2	[270]
D256G	Increased polymerase activity in mammalian cell line	H5N1 backbone with pH1N1 PB2	[271]
T271A	Increase polymerase activity in avian and mammalian cell line	H3N2 (avian)	[272]
		H5N1	[273]
		H7N9	[274]
I292V	Increased polymerase activity in mammalian cell line, increased virulence in mice	H9N2	[275]
	Increased polymerase activity in mammalian cell line	H10N8	[276]
E358V	Decreased virulence in mice	H7N3	[277]
K389R	Increased polymerase activity and replication in mammalian cell line	H7N9	[278]
L339T	Decreased polymerase activity and decreased virulence in mice	H5N1	[279]
K482R	Increased polymerase activity in mammalian cell line	H7N9	[280, 281]
K526R	Increased polymerase activity in mammalian cell line	H5N1	[282]
		H7N9	[282]
M535L	Increased polymerase activity in mammalian cell line	H7N9	[25]
A588V	Increased polymerase activity and replication in mammalian and avian cell lines, increased virulence in mice	H7N9	[276]
		H9N2	[276]
		H10N8	[276]
Q591K	Increased polymerase activity in mammalian and avian cell line, increased replication in mammalian cell line, increased virulence in mice	H5N1	[117, 283]
	Increased polymerase activity in mammalian and avian cell lines	H7N9	[25, 274]
	Increased polymerase activity and replication in mammalian cell line, increased virulence in mice	H9N2	[284]
V598T/I	Increased polymerase activity and replication in mammalian cells, increased virulence in mice	H7N9	[278]
E627K	Increased polymerase activity and replication in mammalian cell line, increase virulence in mice	H4N6	[269]
	Enhanced polymerase activity, increased virulence in mice, contributes to airborne pathogenicity of IAVs in ferrets and contact transmission in guinea pigs. Decreases polymerase activity and replication in avian cell lines. Decreases virulence in chickens.	H5N1	[15, 100, 101, 106–108, 116, 257, 271, 285–289]
	Increased polymerase activity in mammalian cell line, increased virulence in mice	H6N1	[103]
	Increased polymerase activity in mammalian cell lines, increase virulence in mice	H7N7	[102, 104]
	Increased polymerase activity and replication in mammalian cell lines, increased virulence in mice	H7N9	[102, 105]
	Increased polymerase activity in mammalian cell line, increased virulence in mice	H9N2	[102, 290]
E627V	Increased polymerase activity and replication in mammalian cell lines, increased virulence in mice	H5N1	[117]
K627E	Increased virulence in chickens	H5N1	[291]
D701N	Increased viral replication in mammalian cells and virulence in mice	H1N2	[220]
	Increased polymerase activity, enhanced replication efficiency, increased virulence and contact transmission in guinea pigs, increased virulence in mice	H5N1	[96, 117, 206, 292, 293]
	Increased polymerase activity in mammalian cell line	H7N9	[25, 274]
	Increased polymerase activity in mammalian cell line	H9N2	[102]
D701V	Increased polymerase activity and replication in mammalian cell lines, increased virulence in mice	H5N1	[117]
S714R	Increased polymerase activity and replication in mammalian cell line	H7N7	[94, 102, 124]
	Increased polymerase activity in mammalian cell line	H9N2	[102]
S715N	Decreased virulence in mice	H5N1	[294]
M28I, A274T, K526R, I553V, L607V	Decreased polymerase activity in mammalian cell line	H5N1	[295]
L89V, G309D	Increased polymerase activity in mammalian cell line and increased virulence in mice	H5N1	[296]
L89V, G309D, T339K, R477G, I495V, K627E, A676T	Increased polymerase activity in mammalian cell line and increased virulence in mice	H5N1	[296]
M147L, E627K	Increased polymerase activity in mammalian cell line and virulence in mice	H9N2	[297]
I147T, K339T, A588T	Increased polymerase activity in mammalian cell line and virulence in mice	H5N1	[298]
K526R, E627K	Increased polymerase activity and viral replication in mammalian cell lines, increased virulence in mice	H5N1	[282]
		H7N9	[282]
E627K, D701N	Increased polymerase activity in mammalian cell line	H7N9	[299]
E627K, S714R	Increased polymerase activity in mammalian cell lines	H7N7	[102]
		H7N9	[102]
		H9N2	[102]
E627K, D701N, S714R	Increased polymerase activity in mammalian cells, increased virulence in mice	H9N2	[102]
D701N, S714R	Increased polymerase activity in mammalian cell line, increased virulence in mice	H5N1	[300]
	Increased polymerase activity in mammalian cell lines	H7N7	[102]
	Increased polymerase activity in mammalian cell lines	H7N9	[102]
	Increased polymerase activity in mammalian cell lines	H9N2	[102]
E627K (with HA: H110Y, T160A, Q226L, G228S; PB1: H99Y)	Enable airborne transmissibility between ferrets and contact transmission between guinea pigs	H5N1	[15, 58]

Single mutations are presented first followed by motifs involving multiple mutations. Individual markers and motifs are listed in numerical order for ease of identifying mutation of interest

^a^Mutations/motifs are numbered according to alignments with A/Goose/Guangdong/1/1996 (H5N1)

PB2 also potentially antagonizes the host interferon (IFN) response. A subset of AIVs contain PB2 proteins with an N-terminal mitochondrial targeting signal (MTS) that facilitates import of PB2 into the mitochondrial matrix [66, 67]. Mitochondrial PB2 then antagonizes host IFN production by interfering with the action of mitochondrial antiviral signaling proteins (MAVS) [113, 114]. Disruption of the MTS through a single amino acid substitution at position 9 prevents mitochondrial localization of PB2, heightening host IFN response and attenuating viral virulence [115, 116]. Asparagine (N9) or threonine (T9) at position 9 facilitates import of PB2 into the mitochondrial matrix. N9 is typically present in human seasonal viruses (A/H1N1 prior to 2009, A/H2N2, and A/H3N2). In contrast, AIVs typically have aspartic acid at positon 9 (D9), and AIV PB2s are non-mitochondrial and predominantly found in the nucleus. Experimentally mutating seasonal human influenza viruses to contain the non-mitochondrial D9 heightens production of IFN-β and attenuates pathogenicity in mice [115]. Conversely, mutating avian A/H5N1 to contain N9 increases pathogenicity in mice, though specific cellular localization was not investigated [116].

### Polymerase basic protein 1 (PB1; Table 4) and polymerase protein (PA; Table 5)

Mutations in PB1 and PA affect viral replicative capacity and polymerase activity by altering the affinity between components of the RdRP [86–92]. A number of single amino acid substitutions increase AIV virulence in mice, PB1: K577E, D622G; PA: V63I, T97I, K142N/E, K356R, S421I, R443K, and K615N. Of these mutations PB1: N105S, K577E and PA: T97I, K356R also increase AIV polymerase activity in mammalian cell lines at 33 °C (a number of mutations were only tested at 37 °C) [117–119]. The mechanism behind changes in viral replication and virulence caused by these substitutions is not always clear. PA substitutions at positions 63, 97 and 142 could hinder RdRP cap snatching by reducing cleavage of host mRNA caps. Interestingly, four substitutions in PA at positions 142, 147, 171 and 182 that are associated with low polymerase activity of AIVs can drive the emergence of compensatory mutations in other RdRP proteins, as demonstrated for the emergence of PB2 E627K in A(H7N9) viruses [120].

**Table 4:** Experimentally verified molecular markers/motifs in the polymerase basic protein 1 (PB1; segment 2) gene of influenza virus associated with polymerase activity, replication, virulence, and transmissibility

Protein	Mutation/motif	Phenotype	Subtypes tested	References
PB1	D3V^a^	Increased polymerase activity and viral replication in avian and mammalian cell lines	H5N1	[301]
	N105S	Increase polymerase activity and replication in mammalian cell line, increased virulence in mice	H5N1	[117]
	K207R	Decreased polymerase activity in mammalian cell line	H5N1	[302]
	Y436H	Decreased polymerase activity in mammalian cell line; decreased virulence in ducks, ferrets and mice	H5N1	[302]
	V473L	Decreased polymerase activity and replication efficiency in mammalian cells	H1N1 with PB2, PB1, PA NP from H5N1	[303]
	K577E	Increased polymerase activity and virulence in mice	H9N2	[118]
	V598P	Decreased polymerase activity and replication efficiency in mammalian cells	H1N1 with PB2, PB1, PA NP from H5N1	[303]
	D622G	Increased polymerase activity and virulence in mice	H5N1	[304]
	T677M	Increased polymerase activity in mammalian cell line, decreased replication efficiency, decreased virulence in mice	H5N1	[72]
	S678N	Increased replication in avian and mammalian cell lines	H7N7	[124]
	V3A, N328K, N375S	Decreased replication efficiency and virulence in ferrets	H5N1	[93]
	V473L, P598L	Decreased polymerase activity and replication in mammalian cells	H1N1 with PB2, PB1, PA NP from H5N1	[303]
	H99Y (with HA: H110Y, T160A, G226L, G228S; PB2: E627K)	Airborne transmissible in ferrets	H5N1	[15, 58]
PB1-F2	N66S	Enhanced replication, virulence and antiviral response in mice	H5N1	[171, 305]
	T51M, V56A, E87G	Decrease polymerase activity, replication and virulence in ducks	H5N1	[173]

Single mutations are presented first followed by motifs involving multiple mutations. Individual markers and motifs are listed in numerical order for ease of identifying mutation of interest

^a^Mutations/motifs in both PB1 and PB1-F2 proteins are numbered according to alignments with A/Goose/Guangdong/1/1996 (H5N1)

**Table 5:** Experimentally verified molecular markers/motifs in the polymerase (PA; segment 3) gene of avian influenza viruses associated with polymerase activity, replication, virulence, and host inflammatory response

Protein	Mutation/motif	Phenotype	Subtypes tested	References
PA	S37A^a^	Increased polymerase activity in mammalian cell line	H7N9	[280]
	A37S	Decreased polymerase activity in mammalian cell line	H7N7	[306]
	V63I	Increase polymerase activity and enhanced replication in mammalian cell line, increased virulence in mice	H7N7	[306, 307]
	T97I	Increased polymerase activity and replication in mammalian cell line, increased virulence in mice	H5N1	[117]
		Increased polymerase activity in mammalian cell line and enhanced replication in mice	H5N2	[308]
		Increased polymerase activity in mammalian cells	H6N1	[103]
	K142N/E	Increased virulence in mice	H5N1	[116]
	K158R	Increased polymerase activity in mammalian cell line	H5N1	[301]
	P190S	Decreased virulence in mice	H7N3	[277]
	K356R	Increase polymerase activity and enhanced replication in mammalian cell line, increased virulence in mice	H9N2	[119]
	N383D	Increased polymerase activity in mammalian and avian cell lines	H5N1	[121, 122]
	Q400P	Decreased virulence in mice	H7N3	[277]
	N409S	Increased polymerase activity and replication in mammalian cell line	H7N9	[280]
	S421I	Increased virulence in mice	H5N1	[116]
	R443K	Increased virulence in mice	H5N1	[267]
	K497R	Increased polymerase activity in mammalian cell line	H7N9 (human isolate)	[281]
	T515A	Decreased polymerase activity in mammalian cell line, decreased virulence in ducks	H5N1	[302]
	K615N	Increased polymerase activity in mammalian cell line and increased virulence in mice	H7N7	[94, 124]
	A343S, D347E	Increased polymerase activity in mammalian cell line, increase virulence in mice	H5N1	[309]
	P103H, S659L	Decreased polymerase activity replication in mammalian cell line, decreased virulence in mice	H7N7	[310]
	S224P, N383D	Increased polymerase activity and enhanced viral replication in duck and mouse cell lines, increased virulence in mice and ducks	H5N1	[121, 122]
	K142R, I147V, I171V, M182L	Increased polymerase activity in mammalian cell line	H7N9	[120]
	V44I, V127A, C241Y, A343T, I573V	Increased replication in mammalian cell line virulence in mice	H5N1	[311]
	S149P, H266R, I357K, S515T	Increased polymerase activity in mammalian cell line	H5N1	[295]
	K356R (with PB2 E627K)	Increase polymerase activity, enhanced replication capacity in mammalian cell line, increased virulence in mice	H9N2	[119]
PA-X	Truncations resulting in loss of PA-X expression	Increased viral replication in mammalian and avian cell lines; increased inflammatory response in mice; increased virulence in mice, chickens, and ducks	H5N1	[121, 177, 178, 182, 183]
		Decreased virulence in mice, inhibited host inflammatory response	H9N2	[183]

Single mutations are presented first followed by motifs involving multiple mutations. Individual markers and motifs are listed in numerical order for ease of identifying mutation of interest

^a^Mutations/motifs in both PA and PA-X proteins are numbered according to alignments with A/Goose/Guangdong/1/1996 (H5N1)

Interestingly, a combination of two mutations in PA, S224P and N383D, increase polymerase activity of a A/H5N1 subtype virus and enhance viral replication in both mammalian and avian cell lines [121, 122]. This dual mutation also increases A/H5N1 AIV virulence in both mice and ducks. The mutations act in synergy, N383D increases AIV polymerase activity alone while the combination of S224P and N383D significantly increases AIV virulence. This combination differs from previously mentioned mutations as single point mutations in polymerase proteins typically affect viral virulence in mammalian species, with minimal changes in the polymerase activity, replication or virulence in avian species. Some mutations reportedly increase polymerase activity in avian and mammalian cell lines (PB2: A588V, Q591K; PB1: D3V, S678N). However, in these reports, only murine models were utilized to test AIV virulence.

### Nucleoprotein (NP; Table 6)

The NP protein encapsulates viral genomic RNA and mediates import into the nucleus to initiate viral replication through nuclear localization signals and the importin-α/importin-β nuclear import pathway. Mutations that affect the functions of NP have not been extensively investigated; however, substitutions I41V, R91K, R198K, E210D, K227R, K229R, N319K, E434K, K470R enhance polymerase activity in mammalian cell lines [53, 94, 111, 123]. These mutations do not always increase AIV virulence and the mechanism behind the increase in viral replication is not always clear. One particular mutation, N319K, improves A/H7N7 viral replication in mammalian cells by enhancing the interaction between NP and importin-α isoforms [94, 111, 124]. M105V, I109T, and A184K enhance viral replication and increase AIV virulence in chickens [125–127]. Interestingly, the M105V mutation may be involved in spillover adaption from ducks to chickens as M105V affects viral replication in embryo fibroblasts from chickens but not from ducks [127].

**Table 6:** Experimentally verified molecular markers/motifs in the nucleoprotein (NP; segment 5) gene of influenza virus associated with polymerase activity, virulence, and transmissibility

Mutation/motif	Phenotype	Subtypes tested	References
I41V^a^	Increased polymerase activity in mammalian cell line	H7N9	[299]
K91R	Decreased polymerase activity in mammalian cell line	H5N1	[123]
M105V	Increased virulence in chickens	H5N1	[126, 127]
I109T	Increased polymerase activity and viral replication in chickens (but not ducks), increased virulence in chickens	H5N1	[126, 127]
A184K	Increased replication in avian cells and virulence in chickens, enhanced IFN response	H5N1	[125]
K198R	Decreased polymerase activity in mammalian cell line	H5N1	[123]
E210D	Increased polymerase activity in mammalian cell line	H7N9	[299]
K227R	Increased polymerase activity in mammalian cell line	H5N1	[123]
K229R	Increased polymerase activity in mammalian cell line	H5N1	[123]
N319K	Increased polymerase activity and replication in mammalian cell line	H7N7	[94, 111]
E434K	Increased polymerase activity in mammalian cell line	H9N2	[53]
K470R	Increased polymerase activity and replication in mammalian cell line, increased virulence in mice	H5N1	[123]
Q357L (with PB2: E627K)	Increased virulence in mice	H5N1	[116]
E434K (with HA: Q227P, D375E)	Enhanced contact transmission in guinea pigs	H9N2	[53]
E434K (with HA: Q227P, PB2: D195N)	Enhanced contact transmission in guinea pigs	H9N2	[53]
R99K, S345N (with HA: H110Y, T160A, Q226L, G228S; PB2: E627K; PB1: H99Y, I368V	Airborne transmissible in ferrets	H5N1	[15]

Single mutations are presented first followed by motifs involving multiple mutations. Individual markers and motifs are listed in numerical order for ease of identifying mutation of interest

^a^Mutations/motifs are numbered according to alignments with A/Goose/Guangdong/1/1996 (H5N1)

### Non-structural proteins 1 and 2 (NS1/NS2; Table 7)

Influenza virus genomic segment 8 encodes for three viral proteins: NS1, NS2 (Nuclear Export Protein; NEP), and NS3. The NS3 protein was only recently identified and its function remains largely unknown, therefore, mutations in this protein are not included in the inventory [128]. The NS1 protein performs multiple functions that affect AIV replication and virulence (reviewed in [129]). Typically 230 amino acids in length, NS1 ranges between 202 and 238 amino acids in length as the C-terminus is frequently truncated or, occasionally, elongated [130]. Structurally, NS1 proteins contain an RNA-binding domain (RBD) at the N-terminus with a flexible linker region connecting the RBD to a C-terminal effector domain (ED). The RBD and ED are functional domains that mediate the associations between NS1 and interacting partners. NS1 interacts with the vRNP complex, specifically NP and PA, as well as a number of proteins involved in cellular signaling pathways, the host antiviral response, and nuclear/cytoplasmic trafficking or translation of mRNA. Overall,NS1 is the major viral antagonist of the host IFN response and plays a role in host cell shutoff and viral replication [129].

**Table 7:** Experimentally verified molecular markers/motifs in the non-structural protein (NS; segment 8) gene of influenza virus associated with replication, virulence, pathogenicity, and antiviral response

Protein	Mutation/motif	Phenotype	Subtypes tested	References
NS1	P42S^a^	Increased virulence and decreased antiviral response in mice	H5N1	[131]
	D74N	Enhanced replication in mammalian cells and pathogenicity in mice	H7N1 backbone with H5N1 NS	[312]
	80–84 deletion	Increased virulence in chickens	H1N1 (avian)	[313]
		Increased virulence in chickens and mice	H1N1 backbone with H5N1 HA, NA and NS	[134]
		Increased virulence in swine	H1N1 backbone with H5N1 NS	[314]
	Y84F	Decreases replication in mammalian cells and enhances interferon response	H1N1 with H5N1 NS	[132]
	D92E [D87E]^b^	Increased virulence in swine and mice	H1N1 backbone with H5N1 NS	[314, 315]
		Increased virulence in chickens and mice	H1N1 backbone with H5N1 HA, NA and NS	[134]
	I106M [I101M]	Increased viral replication in mammalian cells virulence in mice	H1N1 with all internal genes from H7N9	[316]
	C138F	Increased replication in mammalian cells, decreased interferon response	H5N1	[317]
	V149A	Increased virulence and decreased interferon response in chickens	H5N1	[318]
	L103F, I106M [L98F, I101M]	Increased virulence in mice	H5N1	[319, 320]
	N205S (with NS2: T47A)	Decreased antiviral response in ferrets	H5N1	[133]
	G210R (with: NS2 M51I)	Decreased antiviral response in ferrets	H5N1	[133]
	P3S, R41K, D74N	Enhanced replication in mammalian cells and pathogenicity in mice	H7N1 backbone with H5N1 NS	[312]
	R38A, K41A	Decreased replication in mammalian and avian cell line	H7N1	[321]
	K55E, K66E, C138F	Enhanced replication in mammalian cells, decrease IF response	H5N1	[317]
	222–230 deletion	Increased replication in mammalian and avian cell lines	H5N1	[138]
	225–230 deletion	Increased viral replication in avian cell line	H7N1	[130, 322]
		No impact on viral replication in avian cell lines	H7N1	[140]
	^227^ESEV^230^ (PDZ domain)	Increased virulence in mice	H1N1 pdm09 virus with ‘avian’ PDZ motif	[323]
		Decreased viral replication in mammalian and avian cell lines	H5N1	[138]
		Increased viral replication and virulence in mice, decreased viral replication in human and duck cell lines	H7N1	[137]
	^227^RSKV^230^ (PDZ domain)	Increased viral replication in human and duck cell lines but no effect in murine cells	H7N1	[137]
	230–237 elongation	Increased replication and inflammatory cytokine production in chickens	H9N2	[324]
NS2/NEP	M16I	Increased polymerase activity in mammalian cell line	H5N1	[325]
	M16I, Y41C, E75G	Increased polymerase activity in mammalian cell line	H5N1	[325]
	T47A (with NS1: N205S)	Decreased antiviral response in ferrets	H5N1	[133]
	M51I (with NS1: G2010R)	Decreased antiviral response in ferrets	H5N1	[133]

Single mutations are presented first followed by motifs involving multiple mutations. Individual markers and motifs are listed in numerical order for ease of identifying mutation of interest

^a^Mutations/motifs in NS1 and NEP are presented with numbering relative to A/goose/Guangdong/1/1996 (H5N1)

^b^The CDC molecular inventory numbers NS1 mutations relative to A/Vietnam/1203/2014 (H5N1) which contains an NS1 deletion. For entries from the CDC inventory that uses alternate numbering, the numbering according to A/Vietnam/1203/2014 (H5N1) is included in brackets

Single amino acid substitutions, deletions, and C-terminal truncations in NS1 affect AIV replicative capacity and pathogenicity. Point mutations in NS1 decreasing the host antiviral response (in chickens, ferrets or mice), include: P42S, F89Y, V149A, N200S (with NS2: T47A—see below) and G205R (with NS2: M51I—see below) [131–133]. Deletions and C-terminal truncations of NS1 occur commonly. Contemporary A/H5N1 subtype viruses have five amino acids deleted from position 80 to 84 (relative to A/goose/Guangdong/1996) associated with an increase in virulence in mice and chickens [134]. C-terminal truncations result in deletion of the NS1 PDZ domain, a four amino acid motif that modulates protein–protein interactions with PDZ proteins important for cellular signaling pathways [135]. Human influenza viruses typically contain the PDZ motifs RSKV or RSEV whereas avian viruses have ESEV or EPEV [136] and the influence of PDZ motifs on viral phenotype is host and strain specific. The “avian” ESEV motif decreases viral replication in human and duck cell lines compared to the “human” RSKV motif, whereas these substitutions do not affect virulence in chickens [137, 138]. Overall, the PDZ domain does not appear to be a major determinant of AIV pathogenicity. Indeed, PDZ deletions in the 2009 pandemic A/H1N1 virus as well as in avian A/H5N1 and A/H7N1 subtype AIVs do not significantly affect viral virulence [139, 140].

The NS2 protein, also referred to as the nuclear export protein (NEP) [141], regulates several factors, including: transcription and translation of viral products [142]; nuclear export of vRNP complexes [143]; and viral budding from host cells [144, 145]. NS2 regulates viral polymerase activity, enhancing the synthesis of viral cRNA, vRNA, and, in some cases, mRNA [142, 146]. Very few NS2 mutations that affect viral fitness and host adaptation are characterized in AIVs. Adaptive mutations described in a human A/H5N1 isolate include M16I that, individually or in combination with Y41C and E75G, increase viral polymerase activity in mammalian cell lines [146, 147]. Additionally, two combinations of NS1 and NS2 mutations, T47A with NS1 N200S and M51I with NS1 G205R, decrease the host antiviral response in ferrets [133]. However, the effect of these mutations on AIV pathogenicity is either negligible or unclear [146, 147].

### Matrix protein (MP; Table 8)

Influenza virus genomic segment 7 (MP) encodes for the matrix 1 and 2 proteins (M1 and M2, respectively). M1 is located beneath the viral envelope where it associates with the lipid membrane and viral RNPs. This interaction needs to be ablated for viral RNP to enter the host cell cytoplasm [148, 149]. In the nucleus, M1 associates with vRNPs and mediates export into the cytoplasm [149]. M1 is also involved in viral assembly and budding [150]. M2 is a transmembrane protein present on the surface of influenza virions that acts as an ion channel, acidifying endosomes and contributing to the release of viral RNP into the cytoplasm [151]. The cytoplasmic domain of M2 is also involved in viral genome packaging for progeny viruses [152, 153]. Three more M proteins have also recently been identified: M3, M4 and M42 [154]; however, the functional roles of these proteins are not well established and currently no mutations have been shown to affect viral fitness so they are not included in this inventory.

**Table 8:** Experimentally verified molecular markers/motifs in the matrix (M; segment 7) gene of influenza virus associated with virulence and antiviral resistance

Protein	Mutation/motif	Phenotype	Subtypes tested	References
M1	N30D^a^	Increased virulence in mice	H5N1	[156]
	I43M	Increased virulence in mice, chickens and ducks	H5N1	[157]
	T215A	Increased virulence in mice	H5N1	[156]
M2	L26F	Increased resistance to amantadine and rimantadine	H5N1	[159, 160, 326]
	I/V27A/T/S	Increased resistance to amantadine and rimantadine	H5N1 H5N2	[159–161, 326, 327]
	A30V/T/S	Increased resistance to amantadine and rimantadine	H5N1 H5N2 H7N2	[161, 326, 327]
	S31N/G	Increased resistance to amantadine and rimantadine	H5N1 H5N2 H9N2	[160, 161, 326–330]
	G34E	Increased resistance to amantadine and rimantadine	H5N1	[159]

Single mutations are presented first followed by motifs involving multiple mutations. Individual markers and motifs are listed in numerical order for ease of identifying mutation of interest

^a^Mutations/motifs in M1 and M2 proteins are presented with numbering relative to A/goose/Guangdong/1/1996 (H5N1)

Only a small number of mutations in segment 7 are associated with host adaptation. All occur in the region encoding the M1 protein. Four mutations, namely N30D, I43M, T139A and T215A, increase the virulence of A/H5N1 subtype AIVs in mice [155–157]. I43M also increases virulence in chickens and ducks; however, the underlying mechanisms remain unclear [157]. The M2 protein is an important target of antiviral compounds and mutations in this region contribute to antiviral resistance phenotypes. Adamantanes (amantadine and rimantadine) block the M2 ion channel and inhibit early stages of virus replication. This class of drugs is no longer recommended against seasonal human influenza as these viruses display a high degree of adamantine resistance. In addition, adamantine resistance is increasing in AIVs globally, including subtypes of major concern, such as A/H5 and A/H7 [158]. Well-known mutations associated with adamantane resistance in M2 include: L26F, V27A, A30V/T/S, S31N/G, and G34E [159–161].

## Auxiliary proteins

### PB1-F2 (Table 4)

PB1-F2 is an auxiliary protein expressed in a majority of influenza A viruses and produced from a + 1 alternate reading frame of PB1 [162]. Full-length PB1-F2 is approximately 90 amino acids in length, with frequent variation by truncation. Overall expression and length of PB1-F2 affect influenza virus pathogenicity in both a host and strain dependent manner [163]. PB1-F2 induces host cell apoptosis, antagonizes the host antiviral innate immunity, enhances the production of pro-inflammatory cytokines, and affects viral polymerase activity (reviewed in [164]). Pro-apoptotic activity of PB1-F2 has been reported in human seasonal A/H1N1 influenza viruses but not in A/H5N1 subtype AIVs [165]. The association between PB1-F2, mitochondrial-associated proteins, and cellular factors also inhibits antiviral responses and enhances production of pro-inflammatory cytokines. Interaction of PB1-F2 with MAVS, TBK1 and IRF3. PB1-F2 inhibits the action of TBK1 and IRF3, downregulating host production of type 1 interferon [166, 167]. Additionally, interaction between PB1-F2 and MAVS enhances TRAF6-mediated NF-kB activation, promoting the production of pro-inflammatory cytokines [168, 169]. In A/H5N1 subtype AIVs and human pandemic influenza viruses from 1918, 1957 and 1968, expression of full-length PB1-F2 heightens the inflammatory response to infection in mice, rendering them more susceptible to secondary bacterial pneumonia [170].

Only a few PB1-F2 mutations in AIVs subtypes affect viral pathogenicity. Truncation of the A/H5N1 PB1-F2 protein increases pathogenicity in mice [165, 171], but complete deletion of A/H5N1 PB1-F2 does not significantly alter viral virulence [163, 171]. Perhaps the best-known PB1-F2 mutation, N66S, inhibits host interferon production, increasing pro-inflammatory cytokine responses. However, these effects remain strain and host specific. For example, N66S in A/H5N1 subtype AIV enhances viral replication and pathogenicity in mice but not ducks [171]. Indeed, the few reports that analyze the effect of PB1-F2 expression in avian species show viral attenuation. PB1-F2 expression in A/H5N1 and A/H9N2 subtype AIVs decreases viral pathogenicity in chickens, possibly through inducing the host immune response earlier in chickens compared to mice [172]. Additionally, a combination of mutations T51M/V56A/E87G in PB1-F2 decrease viral polymerase activity, replication, and virulence in mallard ducks [173].

### PA-X (Table 5)

PA-X is a fusion protein comprised of 191N-terminal amino acids (including the endonuclease domain) and 61 amino acids from the C-terminus from segment 3 (PA) formed as the result of ribosomal frameshifting [174]. PA-X has plays a role in host shutoff [174–176], modulating the host immune response [174, 177–179], viral polymerase activity, viral replication, viral induced apoptosis, and virulence (reviewed in [180]). Approximately 75% of all influenza virus isolates possess a full-length PA-X sequence, with the remaining 25% expressing PA-X truncations. These truncations of up to 41 amino acids in length most commonly occur in the PA-X C-terminus and are most commonly found in the 2009 pandemic A/H1N1, canine A/H3N2 and A/H3N8, equine A/H7N7, and bat influenza viruses. However, they also occur in some A/H5N1 and A/H9N2 subtype AIVs [181]. Overall, the function of PA-X appears to be subtype specific. In A/H5N1 subtype AIV, loss of PA-X expression increases viral replication, host inflammatory response, and virulence in mice, chickens, and ducks [177, 178, 182]. In contrast, loss of PA-X in the A/H9N2 subtype limits the host inflammatory response and decreases virulence in mice [183].

## Limitations and usage considerations of this molecular inventory

As stated in the methodology, this inventory was not intended as an exhaustive list so several caveats and limitations need to be considered when utilizing these tables. First, the inventory only includes experimentally validated (in vitro or in vivo) markers/mutations. As stated previously, several studies utilize in silico approaches to predict molecular markers of viral adaptation to humans [22–26]. This work is extremely valuable and findings from these computational studies guide future research. Indeed, novel machine learning approaches have helped to identify novel markers and amino acid positions in human and avian influenza strains associated with pandemic potential [184, 185]. Further work on these novel mutations should be conducted in vitro and in vivo. Second, as stated in the text, changes in biological characteristics for specific point mutations and motifs are sometimes only associated with specific viral subtypes or hosts, so caution should be made when generalizing findings for novel strains or hosts. Finally, consideration must be made for viral sequences themselves. Influenza viruses, like many other RNA viruses, replicate as a quasispecies, allowing for rapid changes in diversity and viral adaptation in response to environmental pressures [186]. The majority of molecular markers/motifs described in these studies come from consensus sequences which may not adequately reflect the entire population of viruses in a given sample. With the continual advancement of Next Generation Sequencing (NGS) and bioinformatics we are better able to understand how minority variants could contribute to pandemic potential of influenza strains. Indeed, recent work by Welkers et al. using NGS on human respiratory A/H5N1 samples identifies multiple single amino acid variants in all three polymerase subunits. In vitro analysis of these markers shows substantial increases in polymerase activity [187]. Therefore, we must move towards consideration of the influenza viral quasispecies as a whole when considering the dynamic evolutionary and adaptive pathways/processes and the meaning and predictive power for zoonotic and/or pandemic potential.

## Discussion/conclusions

Since the discovery of the Goose/Guangdong-lineage A/H5 viruses in China in 1996, HPAIV of the A/H5N1 subtype have spread globally, causing numerous outbreaks in wild birds and poultry. Overall, 861 confirmed human A/H5N1 infections have been reported with a case fatality rate of 52.8% [188]. In addition, newly emerged and emerging AIVs with zoonotic potential continue to appear, including subtypes A/H7N9, A/H7N4, A/H5Nx, A/H9N2, A/H10N7, and A/H10N8 [189–193]. As of June 2019, there have been 1,568 human A/H7N9 cases reported with 616 deaths (CFR: 39%) [194]. There have been 23 human cases of A/H5N6 infection reported from 10 different provinces across mainland China, of which 15 were fatal (CFR 65.2%) [195]. A single human non-fatal infection with an A/H7N4 subtype virus occurred in an elderly woman in Jiangsu, China in December 2017 [192, 196]. This virus is antigenically distinct from formerly circulating A/H7 strains, and, concerningly, appears to be spreading across Southeast Asia, continually reassorting with other viruses in the region [197, 198]. To date, none of the novel A/H7N4 viruses contains known amino acid mutations that confer adaptation of AIV to humans (e.g., PB2 627/701 or HA 186/226/228) or antiviral resistance. However, A/H7N4 isolated from Cambodia does contain the M gene amino acid mutations N30D and T215A that increase pathogenicity of A/H5N1 virus in mice [198]. Due to the antigenic differences between the A/H7N4 viruses and other H7 lineages, including the A/Anhui/1/2013-like A/H7N9 lineage, the continual spread, and risk for human infection, this newly detected A/H7N4 lineage is now in preparation as a candidate vaccine virus for pandemic preparedness [199]. To date, no avian origin influenza viruses can transmit efficiently from human-to-human via aerosols. Given the ongoing spillover of avian influenza viruses into domestic poultry, as well as the risk of human infection and potential for adaptation, continual, vigilant surveillance and risk assessment is vital to combat endemic and emerging AIVs. This inventory provides a list of the currently known, experimentally verified mutations/molecular markers affecting host adaptation in AIVs and can be utilized for molecular characterization and risk assessment of novel AIV strains.
